# Left-Handed Cardiac Surgery Training: How to Attain Proficiency During Residency

**DOI:** 10.1177/15569845241258746

**Published:** 2024-09-12

**Authors:** Eric E. Vinck, Susana Cardona-Bernal, José J. Escobar, Peyman Sardari Nia

**Affiliations:** 1 Department of Cardiothoracic Surgery, Maastricht University Medical Center, Maastricht University, The Netherlands; 2 Department of Cardiac Surgery, Cardio VID Clinic, Pontifical Bolivarian University, Medellín, Colombia

## Introduction

Approximately 10% of the general population and therefore the surgeon population is left-handed (LH), and only 2% to 3% are ambidextrous.^1,2^ Historically, left-handedness in surgery and LH suturing has always been regarded as inferior in relation to right-handedness. Gohil and colleagues mention the 1936 pamphlet by Conway viewing LH as a disease to be eradicated.^2^ LH individuals face a right-handed-tailored world from a young age. In the cardiac surgical field, this is no exception, and training for LH residents is not a smooth process. Although the exact number of LH cardiac surgeons and residents currently practicing or in training is not known, we can assume that with approximately 16,000 cardiac surgeons worldwide (Cardiothoracic Surgery Network), roughly 1,600 (10% LH population) heart surgeons in the world might be LH. Because LH residents are not always overseen by LH cardiac surgeons, many residents are without LH guidance and must adapt to right-handed instructors.^1–3^ Right-handed residents generally have the ease of replicating their instructors whereas southpaw (left-handedness in baseball/martial arts/boxing) residents have to create their own surgical technique without an LH template to follow. Educational resources for LH residents are also scarce. A PubMed search on LH cardiac surgery articles yielded only 10 papers published specific to LH cardiac surgical techniques.^1–8^ Also, cardiac surgery textbooks are illustrated for right-handed surgeons only. Because many LH surgeons were not encouraged to operate with their dominant hand early in their training, some LH cardiac surgeons operate with their right hand resulting from the lack of support and guidance. As a result, there are even fewer LH surgeons using their left hand. Cardiac surgery is already a challenging field requiring stamina, dexterity, manual coordination, and skill. Therefore, encouraging the use of the resident’s dominant hand is imperative to their future success. The idea that LH residents should “just learn to operate with their right hand” implying that any LH resident is also ambidextrous and therefore can easily switch between hands is not logical nor practical. Raising awareness of LH laterality in cardiac surgery and providing solutions and recommendations is imperative for young LH cardiac surgeons.

## Understanding the Challenges of Left-Handedness

Being LH is not performing mirrored cardiac surgery. Many underestimate the learning process and challenges faced by LH cardiac surgery residents. Some authors even recommend developing bilateral dexterity to overcome technical and instrumental difficulties; however, nondominant dexterity can only be partially developed.^1–4^ Although LH surgeons do have situational ambidexterity, this ability should not be universally expected. Being a southpaw is not a question of choice; rather, it is a combination of cerebral architectural functionality and hemispheric laterality. Handedness resulting from this cerebral design is reflected in an LH person’s everyday activities, from brushing their teeth, writing, playing musical instruments, and sports. As the classic adage says, “We do not only write differently, we also think differently.” One common mistake made by LH residents in response to the pressure of performing well is attempting to convert to right-handedness.^1–4^ This puts the LH resident at a disadvantage against true right-handed colleagues, delaying LH surgical skill development and surgical independence (Table 1). This rule may not apply to true ambidextrous surgeons because they can maneuver around their dexterity and switch accordingly. One obstacle during residency is acquiring LH surgical instruments. Although centers should provide LH instruments, LH residents should develop skills using right-handed instruments. Attempting to obtain LH cardiac surgical instruments is many times futile, and residents should not rely on these tools as they will not always be available. Life is easier for residents with access to LH instruments. However, in many emergency cases, right-handed instruments might be more rapidly available. Waiting for your LH instruments can jeopardize patient safety in emergency situations.^1–5^ During training, an LH resident’s surgical confidence and opportunity depends greatly on the support from instructors and cannot be overstated.

**Table 1.** Recommendations for Left-Handed Cardiac Surgery Residents.

Number	Recommendation	Advice	Take into account
1	Be true to your dexterity	Do not attempt to become right-handed	Disadvantage—delay in developing left-handed skills
2	Request left-handed instruments but learn to use right-handed instruments	Do not rely completely on left-handed instruments as they will not always be available	You will many times need to perform procedures with right-handed tools—get comfortable with them
3	Use your right hand if surgical geometry allows	Perfect your laterality but take advantage of your other hand as well	Make your own life easier—let your right hand help too—but operate with your left
4	Find a good left-handed instructor	Request a fellowship with a left-handed surgeon known for teaching left-handed techniques	There will always be tips and tricks you can only learn from a left-handed surgeon

## Resident Versus Fellow

The 5-year and 6-year integrated cardiothoracic surgery training programs, such as in the United States, Netherlands, Brazil, and Spain, do not require a prior general surgery residency.^9^ Other countries such as Belgium, Colombia, and traditional tracks in the United States still retain their fellowship-style tracks with a general surgeon degree as a prerequisite.^9^ What does this mean for an LH trainee? During general surgery residency, an LH resident can develop earlier acquaintance to performing surgery with their left hand and skills using right-handed instruments. Upon migrating to cardiac surgery, the resident will already be comfortable using their left hand, making this transition from general to cardiac surgery much smoother. In contrast, residents entering cardiac surgery without prior general surgery training will have to develop acquaintance with the entire scope of surgery from their LH perspective. Some may argue that this applies for right-handed residents as well; although this may be true, entering cardiac surgery with previous extensive experience in the operating room (OR) as a lefty will no doubt make the transition into cardiac surgery swifter and more efficient.

## Interacting With Right-Handed Staff

Right-handed surgeon supervisors should be supportive and understanding toward LH residents as they have to work harder to learn techniques and get the sequences of cardiac surgery just right. Learning from across the table is not that simple because it is not mirror-image learning; hand dominance is switched for LH residents, not the anatomy. LH residents generally learn from right-handed instructors from the left side and then try to perform the technique with their left hand from the right side. This can get frustrating for both the resident and for the right-handed instructor. In the beginning, one possibly challenging and admittingly frustrating part of being an LH resident is being assisted by a right-handed supervisor. This is specifically true for coronary artery bypass surgery (CABG). Therefore, preoperative communication is critical in preventing confusion or suture entanglement during vascular anastomosis. Many times, a right-handed instructor may get frustrated with an LH resident’s dexterity and make multiple attempts to correct their technique.^1–5^ LH trainees have a mental imagery of what to do and how to move their body that may be different from that of the instructor. Communication of moves and anticipated motions between trainee and instructor is key. It is not important if it is backhand or forehand, facing up or facing down, but more importantly that the suture goes through the tissue in an appropriate angle with minimization of tissue torsion and at an appropriate spacing and depth. One recommendation is to explain clearly how the technique will be executed before surgery; therefore, when the anastomosis is performed, the supervisor expects a certain degree of variation from what they are accustomed to. This will prevent uncomfortable surprises or annoyances during the learning process. An unusual but demotivating scenario can arise when an LH cardiac surgery resident is discouraged from using their dominant hand by an LH surgeon who operates with their right hand. This can occur due to discouragement during the instructor’s own residency. Most LH residents already face isolation from LH cardiac surgeons; therefore, finding an LH instructor who encourages LH dominance in cardiac surgery is vital. Personally, half of the LH cardiac surgeons we have met use their right hand primarily. At the end of the day, each LH surgeon is responsible for performing the surgery in their own fashion and to feel comfortable with their technique.

## Communication With Scrub Nurses

One of the most critical processes during cardiac surgery residency is developing excellent communication with the OR team. This is especially true with scrub nurses. Whenever a resident is allowed to perform cardiac surgery, it is crucial that scrub nurses understand the surgeon’s dexterity. Before surgery, going over the entire surgery with the scrub nurse and clearly explaining needle loading preferences, instrument directions, needle drivers, and Castroviejo delivery is an excellent strategy.^1–5^ This will not only make intraoperative communication better but will avoid unnecessary remounting and other interruptions that delay the surgery and cause irritation from the staff.^1^ A recommendation is to always repeat the desired directions of the needle and driver at different parts of the surgery. This will help the scrub nurse get accustomed to LH surgical techniques and hand dynamics in a progressive manner. Depending on the staff, scrub nurses will either have regular exposure to southpaw surgeons or none. Therefore, LH residents should always remember to be patient and understanding toward scrub nurses since many of them are not accustomed to LH surgeons.

## Southpaw Approach to Cardiac Surgery

### Starting Up

Surgeon positioning has been discussed by LH authors in the past. Because of the normal thoracic and cardiac anatomy and general OR setup, it is recommended to perform cardiac surgery from the right side of the patient.^1–5^ This will allow the OR team to maintain a mental and therefore technical structure of the surgery being performed. Also, to ensure comfort during surgery, the anesthesia bar should be placed far enough so as not to interrupt movements or maneuverings of the left elbow.

### Vein Harvesting

Since vein harvesting is usually the first skill residents are allowed to develop, it is critical that LH residents make good use and take advantage of this time to practice and start to develop their LH skills and use right-handed tools. Because vein-harvesting techniques vary so much, we have no specific recommendations.

### Sternotomy

**Fig. 1.**
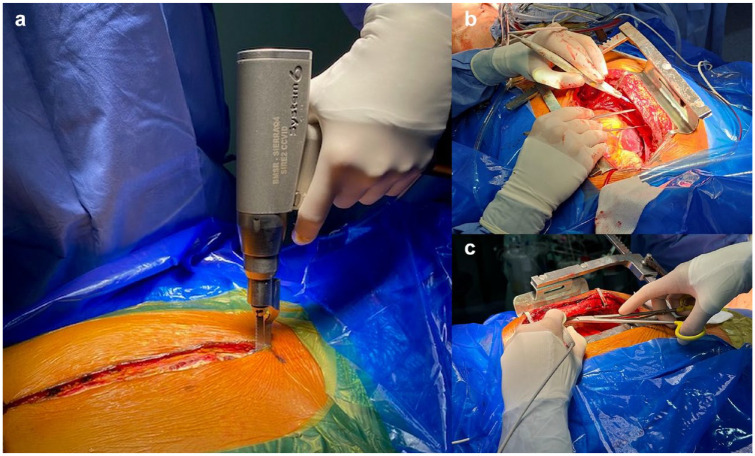
(a) Reciprocating sternal saw with inverted blade for a left-handed sternotomy performed craniocaudally. (b) Left-handed right internal mammary artery takedown. (c) Left-handed left internal mammary artery takedown.

Whether making an LH or right-handed sternal skin incision is not important. This should not matter. However, opening the sternum using your left hand can and does get tricky. The resident can easily perform an LH sternotomy with an oscillating saw. However, for the reciprocating saw, residents have 3 options. First, the sternotomy can be performed with their right hand since switching to the left side of the table may be uncomfortable. Second, the LH resident can perform a caudocranial sternotomy. Third, reciprocating saws have an inverted blade option, which allows residents to perform sternotomies with their left hand craniocaudally; this method is our preferred technique and offers superior saw stability by using the dominant left hand (Fig. 1a).

### Mammary Artery Takedown

Some authors consider it a myth that right internal mammary artery (RIMA) takedown is easier than the left IMA (LIMA) for LH surgeons.^1–5^ We do not believe this to be universally true. Thoracic anatomy, elbow space during takedown, and LH geometry facing the RIMA trajectory all make takedown more ergonomic; remembering that it is not a true mirror image (Fig. 1b). The proximal portions of both mammary artery takedowns are the most challenging as a consequence of the sternal curvature. Due to hand direction and hand positioning, LIMA takedown is more geometric and therefore more ergonomic for right-handed surgeons, while the proximal portion of the RIMA is more ergonomic for LH surgeons. Because the LIMA is harvested more regularly, it may give the illusion that RIMA takedown is not easier. Communication with the scrub nurse during this part of the CABG is important so that it is clear which hand you prefer to have the clips passed to. Keeping the electrocautery in your left hand while switching between forceps and metallic clips with your right hand is an excellent option. This approach does not delay the procedure and is comfortable for scrub nurses since they do not have to reach across to pass the instruments to your left hand (Fig. 1c).

### Purse-String and Cannulation

As long as the anesthesia bar is far enough not to impede left elbow movements, the cannulation process can be done without difficulty. For the LH resident, making the purse-string sutures is much easier with a Castroviejo needle holder unless LH instruments are available. Although Castroviejo needle holders come in both left and right curved versions, the surgeon can simply flip the needle holder over adapting it to the left hand. During this portion of the surgery, many parts including cannulation can be performed bimanually because they do not require too many fine movements. However, purse-string placements should be performed with your dominant hand to avoid taking inaccurate or unnecessarily deep bites.

### Coronary Anastomosis

It is important to remember that there is no absolute correct way to perform coronary anastomoses; practice different approaches to see which best suits you until you have established your own technique. Remember that although certain portions of cardiac surgery can be performed bilaterally, this delicate and precise part requires the dominant hand; therefore, do not allow yourself to be pressured into using your right hand unless you have ambidextrous abilities. The most critical portion of an LH approach to CABG is determining the initial suture placement and direction of the coronary anastomosis. We recommend starting proximal (surgeon’s side) of the coronary artery and suturing clockwise around the heal first. Then, continue distally toward the toe and again proximally so that the knot is tied on the surgeon’s side. Alternating between left forehand and left backhand-mounted needles is a good approach and strategy for performing LH anastomoses especially for the heel portion (proximal to distal) in a clockwise fashion. This approach seems to work well for both saphenous and mammary conduits, always keeping the heel on the surgeon’s left side. Instructors assisting from the left side of the table must be supportive and understand that this LH approach can be visually uncomfortable for them. Again, communicate clearly with your scrub nurse how you want the needle mounted to avoid delay and adjustments.

### Aortic and Mitral Valve Surgery

**Fig. 2.**
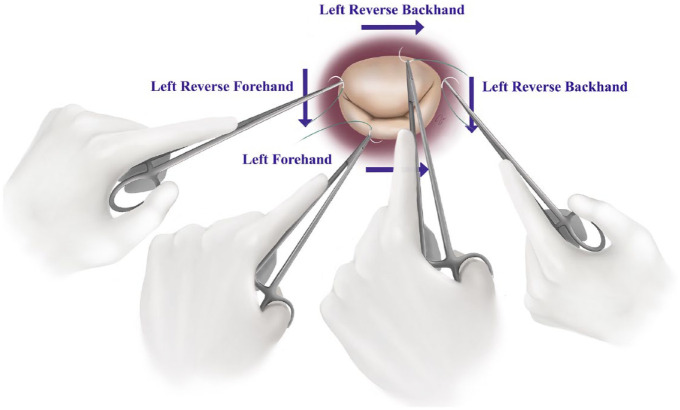
Illustration for left-handed mitral annulus suture placement using a standard needle holder.

**Fig. 3.**
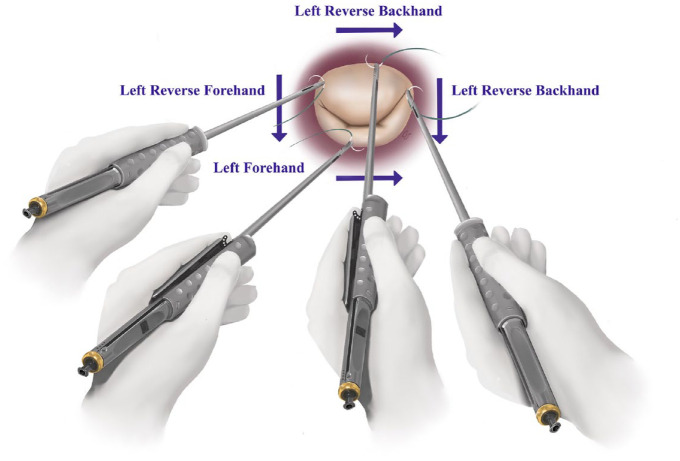
Illustration for left-handed mitral annulus suture placement using a minimally invasive needle holder.

For aortic and mitral valve surgery, the LH resident has a clear strength. From the right side of the operating table, both aortic and mitral valve geometry faces the left hand. This makes placing annular sutures more comfortable. For the mitral annulus suture placement, left forehand, left reverse backhand, and left reverse forehand can be used (Fig. 2). A left reverse backhand works well for the anterior annulus and the medial commissure, whereas a left forehand approach is ideal for the posterior annulus. A left reverse forehand approach can be used for the lateral commissural area (Fig. 2). The same approach can be applied for minimally invasive and endoscopic techniques (Fig. 3). For the aortic valve, sutures can be placed both with a left forehand and left backhand technique. A left forehand approach is optimal for the right coronary annulus as well as the noncoronary annulus. A left backhand works well for the left coronary annulus.

### Sternal Closure

LH wire holders are hard to find. Also, an option is to stand on the left side of the patient for comfort. Even so, adapting to closing the sternum with the right hand is a good option because releasing the rack-and-pinion of a right-handed wire holder with your left hand can be painful. At our center we close the sternum right-handed and without an assistant, as we do not have LH wire holders.

### When to Use Your Right Hand

There are clear advantages for residents with certain ambidextrous abilities and those who are clearly ambidextrous but primarily LH. The ability to quickly maneuver instruments to accommodate the surgery is an ability that can simplify cardiac surgery tremendously. An example of situational ambidexterity is when there is unexpected bleeding requiring swift hemorrhage control that is better corrected by a right-handed approach. Although the LH cardiac surgery resident should aim to perfect their LH laterality, they should remember that the nondominant hand is an invaluable companion that should be used whenever deemed necessary (Table 1).

## Discussion

In their article, Adusumilli and colleagues found that 90% of surgeons had no mentoring for being LH and 87% were discouraged from surgical specialties for being LH.^10^ Also, 97% never received guidance for being LH during medical school, and 87% of surgeons had no access to LH instruments during training.^10^ Although these numbers are not specific to cardiac surgery, they do offer similar perspectives. Although many deem left-handedness to be a disadvantage, LH surgeons regard their left-handedness as a significant advantage once they initiate independent practice. This is due to the freedom of maneuvering and choosing their own surgical approaches once out of training.^11^ It has also been shown that in laparoscopy, LH residents show less variability during endoscopic performances than their right-handed colleagues.^12^ Sadly, despite the apparent advantages of southpaws, LH surgeons have considered changing specialties and even leaving surgery altogether due to dexterity-related frustrations.^3^ Also, it has been shown that 73% of surgeons have had to remind their staff of their dexterity during surgery, and 28% feel at increased risk of stick injuries during surgery because of their laterality.^3^

LH residents who have had the opportunity to train under southpaw instructors and have been encouraged to hone their LH skills are truly fortunate. For those trainees not so fortunate, seek an LH cardiac surgeon known to teach LH techniques. Request to shadow or ask for a rotation/fellowship to develop or even perfect certain skills. Just as many young surgeons seek fellowships in minimally invasive, robotic, transplant, and other scopes of cardiac surgery, requesting time to train under an LH surgeon can be of great help (LH fellowship). Finally, remember that being a southpaw is an advantage but requires hard work to develop the necessary skills in cardiac surgery. Often times, the obstacles from being LH are underestimated, but so are the advantages. Programs should adapt to LH dominant laterality, encourage LH residents, and allow them to discover their own technique. From the resident standpoint, attitude and behavior toward instructors and OR staff are critical, as these can accelerate or delay your progress. Be patient and remember that attitude is everything during residency.^13^

As cardiac surgery keeps evolving into more minimally invasive techniques and endoscopic approaches, the LH resident and surgeon must be well aware to keep up and understand the importance of continuous growth and developing LH skills within the scope of minimally invasive procedures.^14^ Just as instruments are built for the right-handed, so are many surgical teaching strategies and simulation training devices for minimally invasive cardiac surgical education. As cardiac surgical simulation training keeps evolving, more platforms and more solid evidence will arise, encouraging the need for LH-tailored simulation systems (Fig. 3). Consequently, the continuous emphasis on LH-specific training to reach proficiency for the southpaw surgeon remains. As more LH cardiac surgery papers and online videos become available, LH residents will have more resources to turn to for improving their technique. This will also allow for LH cardiac surgeons to pass down their knowledge and experiences to younger surgeons. Accepting that challenges do exist for the LH cardiac surgery resident is imperative. However, the repeated notion of left-handedness being a “problem” or “setback” in surgery should be abandoned. Left-handedness is an advantage in cardiac surgery just as in sports. However, it does have clear obstacles that require additional work to overcome and achieve proficiency during the training process.

## Conclusions

The world of cardiac surgery and surgical instruments are designed for right-handed surgeons. Yet, LH surgeons have clear advantages. The historical notion of the LH disadvantage should be abandoned as left-handedness can be developed into an invaluable arsenal in cardiac surgery. Simply, there are additional challenges to surpass and greater skill sets to cultivate. It is imperative that the cardiac surgical resident understand that despite the challenges they need to overcome, they should be encouraged to hone their skills.
